# *Stolonicaulon*: A Section-Puzzle within *Marsupella* (Gymnomitriaceae, Marchantiophyta)

**DOI:** 10.3390/plants11121596

**Published:** 2022-06-17

**Authors:** Vadim A. Bakalin, Anna A. Vilnet, Yuriy S. Mamontov, Alfons Schäfer-Verwimp, Yulia D. Maltseva, Ksenia G. Klimova, Van Sinh Nguyen, Seung Se Choi

**Affiliations:** 1 Laboratory of Cryptogamic Biota, Botanical Garden-Institute FEB RAS, Makovskogo Street 142, Vladivostok 690024, Russia; maltseva.yu.dm@gmail.com (Y.D.M.); ksenia.g.klimova@mail.ru (K.G.K.); 2 Laboratory of Flora and Vegetation, Polar-Alpine Botanical Garden-Institute of the Russian Academy of Sciences, Akademgorodok, 18A, Apatity 184209, Russia; anya_v@list.ru (A.A.V.); yur-mamontov@yandex.ru (Y.S.M.); 3 Herbarium, Tsitsin Main Botanical Garden RAS, Botanicheskaya 4, Moscow 127276, Russia; 4 Mittlere Letten 11, 88634 Herdwangen-Schönach, Germany; moos.alfons@kabelbw.de; 5 Institute of Ecology and Biological Resources, Graduate University of Science and Technology, Vietnam Academy of Science and Technology, Ha Noi 10072, Vietnam; vansinh.nguyen@iebr.ac.vn; 6 Team of National Ecosystem Survey, National Institute of Ecology, Seocheon 33657, Korea

**Keywords:** *Marsupella*, Gymnomitriaceae, molecular phylogenetic, Pacific Asia, phytogeography, Marchantiophyta

## Abstract

*Marsupella* sect. *Stolonicaulon* is not speciose and is a commonly neglected section within the genus, which currently includes three species with somewhat similar morphologies (wiry shoots with distanced leaves) and distributions in the mountains of tropical and subtropical regions (SE (Southeast) Asia, the Venezuelan Andes, and the high mountains of SE Brazil). After studying materials that were found to be dissimilar to the “traditional” *Marsupella* that were collected in the last decade by the authors of this article, it was found that these plants belong to three new-for-science species, and all of these species should be included in *Marsupella* sect. *Stolonicaulon*. The newly described species have expanded the boundaries of morphological variability, not only for the section itself, but also for the genus based on two findings: (1) the leaves of *Marsupella* sect. *Stolonicaulon* can be either appressed and entire or spaced and deeply divided (thus, the plants could occasionally be similar to *Cephaloziella* or *Anastrophyllum*); (2) some species of the section possess regular underleaf production. The first discovery of regular underleaves in *Marsupella*, as noted in two of the three newly described taxa, is the main morphological novelty described in this paper. The development of regular underleaves is a presumable relict character that brings *Marsupella* closer to *Nardia*, which was recently transferred to the Gymnomitriaceae and occupies an isolated position within its own subfamily, Nardioideae.

## 1. Introduction

*Marsupella* sect. *Stolonicaulon* (N. Kitag.) Váňa is perhaps the most mysterious and peculiar section within the genus due to its controversial morphological features and southern distribution, strongly contradicting other sections of the genus. Two efforts to make the system of the sect. *Stolonicaulon* more sound were made in the 20th century. The first was by Kitagawa [1] who described *Marsupella stoloniformis* N. Kitag. as well as sect. *Stolonicaulon* by itself. Kitagawa, however, was limited to researching the only known species belonging to the monotypic section. The next advance was made by Schuster [2], who described *Marsupella stoloniformis* subsp. *vermiformis* R.M.Schust. The lack of both fresh and old herbarium materials has prevented research on this section for several years, although there were several other attempts to clarify the systematic classification of *Marsupella* based on a new approach, including molecular genetic studies. The most valuable results in this field were accessed by Vilnet et al. [3] and Shaw et al. [4]. Several species were transferred from *Marsupella* to *Gymnomitrion*, while *Nardia* (previously placed in Jungermanniaceae) was placed within Gymnomitriaceae, but in its own subfamily [5]. Meanwhile, the exhaustive worldwide compendium of Gymnomitriaceae (based mostly on data obtained in the ‘pre-molecular era’) was published by Váňa et al. [6]. The last regional attempt to clarify the *Marsupella–Gymnomitrion* complex in East Asia involving recently collected materials was made by the revision of Korean Gymnomitriaceae [7]. All of these updates made the infrageneric structure of *Marsupella* sound but did not further our understanding of sect. *Stolonicaulon* due to the aforementioned lack of material.

Some newly collected materials belonging to *Marsupella* sect. *Stolonicaulon* that were collected in East and Southeast Asia have permitted scholars to discuss the taxonomy of this section again, such as the discussions by Bakalin et al. [8] in a review of Asian amphi-Pacific *Marsupella*. In summary, the results of the work by Bakalin et al. are as follows: (1) *Marsupella* sect. *Nanocaulon* R.M. Schust. is synonymized under subg. *Stolonicaulon* (the latter name has priority); (2) *Marsupella stoloniformis* subsp. *vermiformis* was raised to the rank of species (*M. vermiformis* (R.M. Schust.) Bakalin et Fedosov) based on robust genetic differences with *M. stoloniformis* s. str.; (3) following the synonymization of sect. *Nanocaulon* with sect. *Stolonicaulon*, *Marsupella microphylla* R.M. Schust. was added to sect. *Stolonicaulon*. Regarding molecular genetics, such research was based (concerning the sect. *Stolonicaulon*) on new sequences from three specimens: two from *M. vermiformis* and one from *M. stoloniformis*. Decisions on the taxonomic position of *M. microphylla* were made on the basis of morphological descriptions, and, of course, these may be questioned. It is worth mentioning that *M. microphylla* is from Venezuela and Brazil and may refer to the section that is confined to the mountains of tropical and subtropical Asia eastward to Melanesia with some reservations.

Continuing the floristic exploration in Southeast and East Asia, the coauthors of the present work independently discovered strange plants, which were occasionally identified under a dissecting microscope or hand lenses as *Cephaloziella* or *Anastrophyllum*. Later, after attempting to understand the systematic position of these plants, these assessments revealed that all of the plants are even representatives of the same section (*Stolonicaulon*) despite strong morphological differences. The revealed diversity of morphotypes significantly refines both the morphological boundaries of the section itself (and partly of the genus as a whole) and forces us to take a different look at the distinguishing morphological criteria of the species within *Marsupella*. As is evident from that noted below, the representatives of sect. *Stolonicaulon* possess a span of morphological variability that is not narrower than that of all other *Marsupella* taxa taken together. The description of new data on the taxonomy of sect. *Stolonicaulon* was the main goal of this work.

## 2. Results

The ITS1-2 and *trn*L-F sequence data were obtained for two specimens from Taiwan, and ITS1-2, *trn*L-F and *trn*G-intron were obtained for specimens from Vietnam. Seven newly generated accessions were deposited in GenBank. The combined alignment of two genomic regions for 33 specimens revealed 1291 character sites, among which 888 positions belonged to ITS1-2 and 403 belonged to *trn*L-F.

**Figure 1:**
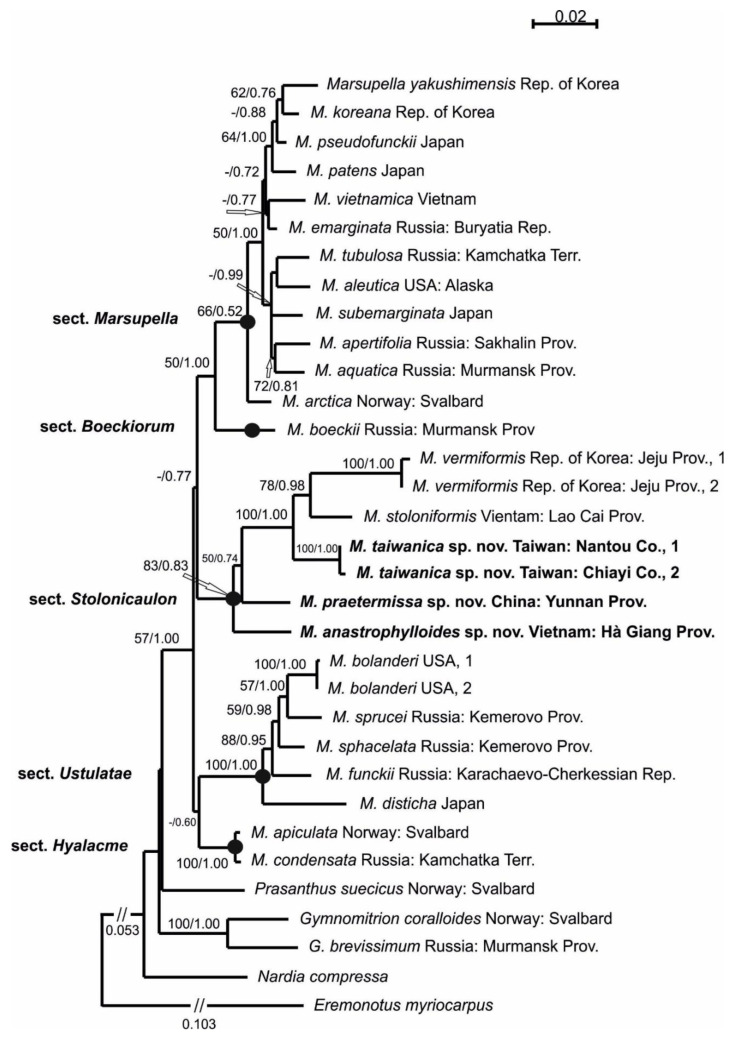
Phylogram obtained in a maximum likelihood calculation for the genus *Marsupella* based on ITS1-2+*trn*L-F dataset. Bootstrap support values of maximum likelihood analysis more than 50% and Bayesian posterior probabilities more than 0.50 are indicated.

The ML analysis resulted in a single tree with an arithmetic mean of the log likelihood of −5676.544929. In the Bayesian analysis, the arithmetic means of the log likelihoods for each sampling run were −5525.73 and −5525.83. The tree topologies that were obtained by both methods became congruent. Figure 1 demonstrates the ML topology with an indication of bootstrap support values (BS) and Bayesian posterior probabilities (PP). The backbone phylogeny of the genus *Marsupella* differs in the position of sections *Hyalacme*, *Ustulatae*, and *Stolonicaulon* compared with the phylogeny that was published by Bakalin et al. [8]. In both cases, some nodes received slight support. This finding may be explained by the influence of different outgroup sampling. The phylogenetic schema obtained previously [8] suggested the position of the section *Stolonicaulon* in the base of the genus *Marsupella* with 1/84 supports. Our estimation revealed that the section *Stolonicaulon* has a sister affinity to the clade, with sections *Marsupella* and *Boeckiorum* supported only in BA with PP = 0.77. Nevertheless, the affinity among the sections remains dubious, but their composition is quite stable. All of the six tested specimens comprised a clade corresponding to section *Stolonicaulon* with the support of BS = 83% and PP = 0.83 (or 83/0.83). The position of this clade in the backbone phylogeny was supported only in BA (-/0.77). Two sequenced Korean specimens of *Marsupella vermiformis* were found to be related to Vietnamese specimens of *M. stoloniformis* (78/0.98), and they were both characterized by long branches. Two specimens from Taiwan were found to comprise a subclade (100/1.00) related to *Marsupella vermiformis* + *M. stoloniformis* (100/1.00). The next divergence belongs to the Chinese specimen that was first published as *M. stoloniformis* by Shaw et al. [4] (50/0.74). The specimen from Vietnam exhibited a basal position in the clade of the section *Stolonicaulon*.

The *p*-distance estimation revealed ITS1-2 variability in two sequenced specimens of *M. vermiformis* that could be incorrect readings (Table 1). Two specimens from remote regions of Taiwan exhibited variability in both ITS1-2 (0.1%) and *trn*L-F (0.3%) loci, and this finding is consistent with the level of intraspecific variability in the genus *Marsupella* [9]. The divergence among taxa varies from 2.5 to 4.4% for ITS1-2 and from 3.9 to 8.9% for *trn*L-F. A 4.6% difference in *trn*G-intron counts between the Chinese specimen that was first published as *M. stoloniformis* and the putative Vietnamese specimen was observed (data not shown in Table 1).

Thus, phylogenetic affinity and the level of sequence divergence revealed the existence of three new science species from the genus *Marsupella* section *Stolonicaulon*: *M. taiwanica*, *M. praetermissa* and *M. anastrophylloides*.

## 3. Discussion

A phylogenetic analysis yielded a topology that was similar to that published by Bakalin et al. [8]. Previously, we wrote [8] (p. 63) the following: “Sect. *Stolonicaulon* is highly distinctive from the other bulk of *Marsupella* in a number of features, including … very rigid shoots, strongly distanced, scale-like leaves appressed to the stem and meso-xerophilous habitat that contradicts to the majority of other *Marsupella*”. This phrase requires clarification. Indeed, all representatives of the section (as it is known today) are small plants, and most of them are wiry. However, their leaves may not be appressed to the stem and they are not highly distanced.

The basal branch to all other members of sect. *Stolonicaulon* is formed by *Marsupella anastrophylloides*, which definitely justifies its name based on its resemblance to small representatives of *Anastrophyllum* or *Sphenolobopsis*, as well as deeply incised bilobed leaves with narrow lobes. This species is the most dissimilar to the other representatives of the section. In this case, the genetic distance correlates well with morphology.

Another well-circumscribed taxon described here is *Marsupella taiwanica*, which is similar to *Cephaloziella* due to incised leaves and small regular underleaves and therefore strongly dissimilar to other *Marsupella*. However, the generative features of this taxon definitely possess the character of other representatives of sect. *Stolonicaulon*, including a long perigynium, which is partially exposed due to relatively short leaves.

Three other species are superficially (under a dissecting microscope) similar to each other morphologically (wiry shoots, distanced leaves, and a stem that is almost as wide as the left shoot). However, these species are not as closely related in the phylogenetic tree as could be suggested based on an initial look. The two species that are most closely related to each other are *Marsupella stoloniformis* and *M. vermiformis*. Their morphological differences have been discussed previously [8]. These differences mainly involve short emarginate to entire leaves and somewhat hygrophilous ecology in *M. stoloniformis* versus mostly bilobed, sharply pointed leaf lobes and somewhat xerophilic ecology in *M. vermiformis*. The third species is similar to both aforementioned species and is described here as *M. praetermissa*. The original specimen (treated here as holotype) was originally confused with *M. stoloniformis* and appeared in the paper by Shaw et al. [4] under the latter name. However, the comparison of this specimen with a type of *M. stoloniformis* (KYO, Mizutani 2788!) revealed that it is not a conspecific of the type. Indeed, *M. praetermissa* is characterized by a morphology that is similar to that of *M. stoloniformis* (distanced, entire-to-shallowly emarginate leaves, and a comparatively thick stem); however, the leaves of this species narrowly spread from the stem, even under dry conditions, which clearly contradicts the always appressed leaves in the type and other known plants of *M. stoloniformis*. The main distinguishing feature is regular, albeit small underleaves that are present in *M. praetermissa*. The latter to a certain extent explains its relationships with *M. taiwanica* in the phylogenetic tree (despite the superficial morphology, the two taxa are strongly dissimilar).

The occurrence of underleaves was not known in *Marsupella* prior to the present work. As currently reported, two described species, *M. taiwanica* and *M. praetermissa*, show regular underleaves. Moreover, it is worth mentioning that *M. anastrophylloides* rarely possess the ability to develop 1–2 subulate underleaves just above the ventral branch (although very irregular), whereas underleaves are completely absent in unbranched shoots. Therefore, in the taxon that is most genetically distanced from others (*M. anastrophylloides*), the presence of underleaves is rare. In contrast, underleaves are regularly noted in the two other taxa (*M. taiwanica* and *M. praetermissa*). In *M. stoloniformis* and *M. vermiformis*, underleaves are completely absent.

The stem cross section also possesses noticeable variation across sect. *Stolonicaulon*. Two earlier known taxa (*M. stoloniformis* and *M. vermiformis*) have no hyaloderm cells (the cells are thick-walled and not—or slightly—different in size from the cells inward in both). *Marsupella taiwanica* is an intermediate variant in this feature. Specifically, its outer cells in the stem cross section are larger than the inner cells and have distinctly thinner walls; however, these cell walls are much thicker than those that are observed in the two remaining taxa. Another extreme (in contrast to *M. vermiformis* and *M. stoloniformis*) is the pair *M. praetermissa* and *M. anastrophylloides*. These two taxa exhibit strongly different leaf morphologies but possess distinct hyalodermis of thin-walled cells that are strongly different from scleroderm cells inward.

Therefore, *Marsupella* sect. *Stolonicaulon* possesses wide variation in several morphological features:

(1) Leaf shape and comparative stem size (with the stem diameter). Leaves may be contiguous to highly distanced, ranging from deeply divided and appressed to the stem to narrowly spreading;

(2) Stem cross-section features. Although all species have thick-walled scleroderm cells and more or less thick-walled cells in the inner part, the outer cells of the stem can be either thick-walled or distinctly thin-walled and form a distinct hyaloderm (although they commonly only slightly differ in size from the inner cells);

(3) Underleaf presence. This feature makes the section unique among *Marsupella*. Regular underleaves are present in *M. praetermissa* and *M. taiwanica*. Underleaves are also present in *M. anastrophylloides* as a rare exception. Underleaves are generally a very unusual characteristic within Gymnomitriaceae. This family in the “old” sense did not include genera with underleaves at all [6]. The only presumable ‘member’ of Gymnomitriaceae with regular underleaves (as long as leaf length!) is monotypic *Paramomitrion* R.M. Schust., which has only been reported in sterile conditions from the only collection that was made by R.M. Schuster in Venezuelan Andes. It is questionable whether *Paramomitrion* belongs to Gymnomitriaceae at all. In the original description of the genus and species, Schuster [2] (p. 138) wrote the following: “I place it in the Gymnomitriaceae, next to *Eremonotus*—but without any conviction that this is the final resting place”. *Eremonotus* clearly belongs to Jungermanniaceae, whereas the molecular-phylogenetic affinity of *Paramomitrion* is not known.

However, the molecular revision of the suborder Jungermanniineae [4] has necessitated the transfer of *Nardia* to Gymnomitriaceae (albeit within the isolated subfamily Nardioideae Váňa) among other things. Thus, Gymnomitriaceae became partly an ‘amphigastrious’ family. The present account has shown that distinct regular underleaves are also possible in the subfamily Gymnomitrioideae in addition to the atavistic underleaves, which were indicated for some species of *Gymnomitrion* [10]. Underleaves are occasionally present in *G. laceratum* (Steph.) Horik. The presence of regular underleaves in *Marsupella* is most likely a plesiomorphic trait, emphasizing its relationship to *Nardia*. In this regard, two species of *Nardia* have been described that are new to science within recent years (*Nardia minutifolia* Furuki and *Nardia grollei* Váňa et D.G. Long) and should be mentioned. Both are characterized by entire leaves that only slightly exceed the width of the stem, and both were not tested genetically. Such species may need to be tested to assess their relationship to *Marsupella* sect. *Stolonicaulon*. However, these species are most likely not related to the genus *Marsupella* sect. *Stolonicaulon*, which is characterized by cell sizes which are much smaller than that noted in both mentioned *Nardia* species.

In addition to morphology, *Marsupella* sect. *Stolonicaulon* shows a peculiar distribution pattern that distinguishes it from other sections of the genus. All representatives of the section are distributed in the mountains in the tropical and subtropical regions of Pacific Asia to Melanesia (the northernmost occurrence of *M. vermiformis* is noted in the mountains of Jeju Island, where the vegetation of the lowlands is subtropical). One species, namely, *Marsupella microphylla* R.M. Schust., is known from the Neotropical Floristic Kingdom (Venezuela and Brazil), but its assignment to sect. *Stolonicaulon* can be questioned. This poorly understood species has not been studied using molecular genetic methods and its systematic position in *Marsupella* is unclear.

## 4. Taxonomic Treatment

*Marsupella anastrophylloides* Bakalin, Vilnet et Maltseva ***sp. nov.***

Description. Plants scattered in the mat of *Kurzia makinoana*, copper and pinkish brownish to purplish, somewhat rigid and very fragile when dry, prostrate to loosely ascending, 300–400 µm wide and 3–15 mm long, rhizomatous base absent. Rhizoids virtually absent. Stem brownish to purplish, branching sparse, ventral, producing normal shoots, ventral merophyte 1–2 cells wide, free of leaves line in dorsal side 0–1 cells wide; cross section orbicular, 7–8 cells high, outer cells hyalodermatic, thin-walled, with small concave trigones, 7–8 µm in diameter to oblong 8–10 × 6–7 µm, inward with distinct scleroderma composed by very thick-walled cells with moderately sized, concave trigones, and then passing into inner cells with thickened walls (although by far not as thick as outer), 10–12 µm in diameter. Leaves contiguous to somewhat distant (never strongly so), transversely inserted and oriented and obliquely spreading, ovate in outline, divided to 3/5–2/3 of leaf length into 2 slightly diverging lobes by V- to gamma-shaped sinus, lobes acute, terminating by 1–2 uniseriate cells, 240–270 × 170–250 µm, widest below sinus. Underleaves absent, rarely occurring as short subulate remnants between 1 or 2 pairs of leaves above origin of ventral branch. Cells in the middle of undivided part of leaf lamina subisodiametric, 8–10 µm in diameter to oblong, 10–20 × 7–13 µm, prominently thick-walled with moderately sized concave trigones, middle lamellae well visible in the trigones, pink to purplish in color, other parts of cell walls pinkish brownish. Cells in lobe middle subisodiametric, 7–10 µm in diameter, apical cells oblong, 8–15 × 6–10 µm, very thick-walled, with visible middle lamella in the trigones those are moderate in size, concave; cuticle virtually smooth throughout, although sometimes indistinctly verrucose along leaf margin. Generative structures unknown.

Holotype: Vietnam, Hà Giang Province, Vi Xuyên District, Cao Bo Commune, Tay Con Linh Range, Tay Con Linh Nature Reserve (22°47′53.0″ N 104°48′33.5″ E), 2296 m a.s.l., south subtropical mountain evergreen forest with large conglomerate cliffy massif, open mesic cliff; 22 March 2020, V.A. Bakalin & K.G. Klimova, V-15-6-20 (VBGI).

Etymology: Its name was given based on morphological similarity to small *Anastrophyllum*.

Ecology: The species was only collected once, therefore, its ecology is incompletely known. It was gathered in a southern subtropical mountain forest over open mesic cliffs as an admixture in the mat of *Kurzia makinoana*. The plants were creeping over *Kurzia*. The same patch contains a sparse admixture of the xeromorphic modification of *Anastrophyllum bidens*.

Comment: Due to its prominent external characteristics, including contiguous obliquely spreading and deeply divided leaves, the species could be confused with all other known *Marsupella*. However, in the sterile state (as it was only collected), the species may be mistaken for depauperate plants of *Anastrophyllum bidens*, from which it differs based on a distinct tint of copper or pinkish pigmentation and distinctly smaller leaf cells (7–13 µm wide, versus 15–20 µm wide in the midleaf) with thick walls, concave trigones (versus trigones large, prominently bulging), and fragile shoots (versus shoots more elastic). Disregarding the copper and pinkish pigmentation, the species somewhat resembles *Sphenolobopsis pearsonii*, but the leaf cuticle is virtually smooth and regular underleaves are absent in *Marsupella anastrophylloides*. The main difference from the latter potentially occurs in generative structures, which are unfortunately unknown.

Given the strong molecular dissimilarity to other taxa and no distantly similar regional taxa, we hypothesized that the species has undergone a long period of isolation and speciation. If so, the expectation of its current distribution is very speculative since the ‘old-existing’ taxon might have a wide original area and be preserved in several restricted areas as a geographic relict.

**Figure 2:**
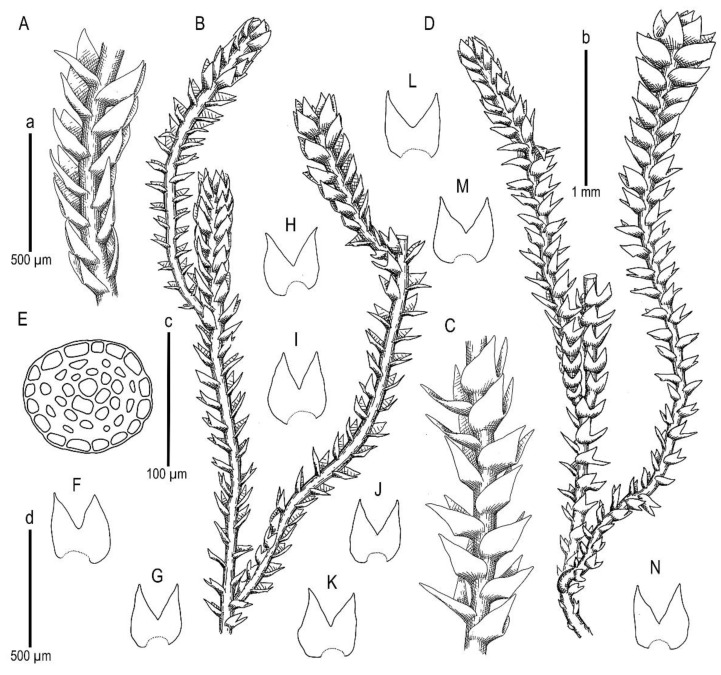
*Marsupella anastrophylloides* Bakalin, Vilnet et Maltseva ***sp. nov.***: (**A**,**B**) plant habit, fragment, dorsal view; (**C**,**D**) plant habit, fragment, ventral view; (**E**) stem cross-section; (**F**–**N**) leaves. Scales: a—500 µm for (**A**,**C**); b—1 mm for (**B**,**D**); c—100 µm for (**E**); d—500 µm for (**F**–**N**). All from Holotype V-15-6-20 (VBGI).

**Figure 3:**
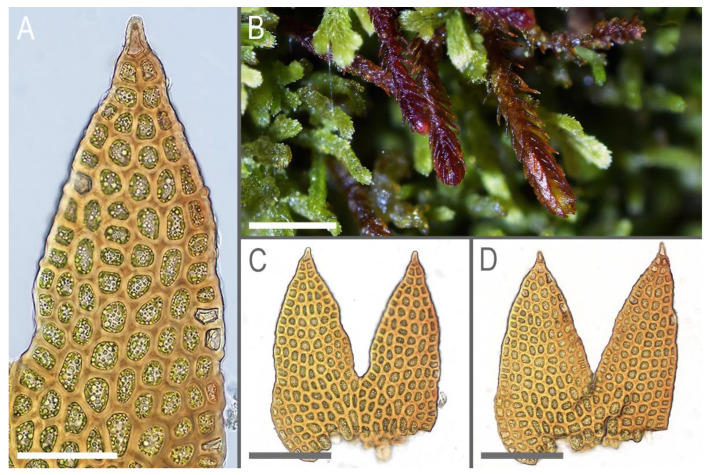
*Marsupella anastrophylloides* Bakalin, Vilnet et Maltseva ***sp. nov***.: (**A**) leaf lobe; (**B**) plant habit; (**C**,**D**) leaves. Scales: 50 µm for (**A**); 1 mm for (**B**); 50 µm for (**C**,**D**). All from Holotype V-15-6-20 (VBGI).

Illustrations in present paper: Figure 2 and Figure 3.

*Marsupella praetermissa* Bakalin et *Vilnet* ***sp. nov.***

Description. Plants brownish to brown, wiry, rigid, forming loose patches with *Riccardia*, *Sphenolobopsis pearsonii* and *Gymnomitrion* (*Apomarsupella* group), 120–150 (170) µm wide and 3–8 mm long. Rhizomatous base is ill-developed. Rhizoids colorless to purplish at the branch base, above only as exception and never in upper half of the well-developed shoot. Stem sparsely ventrally branched in rhizomatous base, cells in dorsal surface subquadrate to 4–6-gonal, ventral merophyte 2 cells wide, dorsal row free of leaves 0–1 cell wide; cross section transversely ellipsoidal to rotundate, 80–110 µm in diameter to 90 × 100–120 µm, outer cells thin-walled (hyalodermatic), 7–8 µm in diameter with small concave trigones, inward suddenly become thick-walled, with visible middle lamella, 6–9 µm in diameter, with large triangular trigones, cell walls of medullary cells slightly thinner, but still very thick. Leaves transversely inserted and oriented, obliquely spreading, somewhat concave-canaliculate, sheathing the stem, leaf apex entire to emarginate, well developed leaves longer than wide, 100–170 × 90–150 µm. Underleaves vestigial, although highly regular, spathulate to ovate, (1–)2 cells wide and 2–3 cells long, rarely subulate 1 cell wide and 3 cells long. Midleaf cells subisodiametric to oblong, 6–8 µm in diameter to 7–10 × 6–8 µm, thin-walled or with thickened walls, trigones moderate in size, concave; cells along leaf margin slightly larger and with thicker and deeper colored walls than just inward, 8–10 µm in diameter or oblong (elongate along margin), 8–10 × 6–8 µm; cuticle virtually smooth throughout. Generative structures unknown.

Holotype: China, Yunnan Province, Gongshan County, Cikai Xheng, east slope of Gaoligong Shan, Nu Jiang (Salween) catchment, Yipisaka Lake at head of Pula He valley, 2.2 km SSE of tunnel (27°45′12.4″ N 98°27′36.4″ E), 3455 m a.s.l., granitic cliff on alpine lake shore; on small boulder under shady dripping cliff, 12 August 2006, D.G. Long, Long 35,742 (E, duplicate in VBGI).

Etymology: the name ‘*praetermissa*’ is translated as ‘overlooked’ from Latin due to its superficial similarity to *M. stoloniformis*.

**Figure 4:**
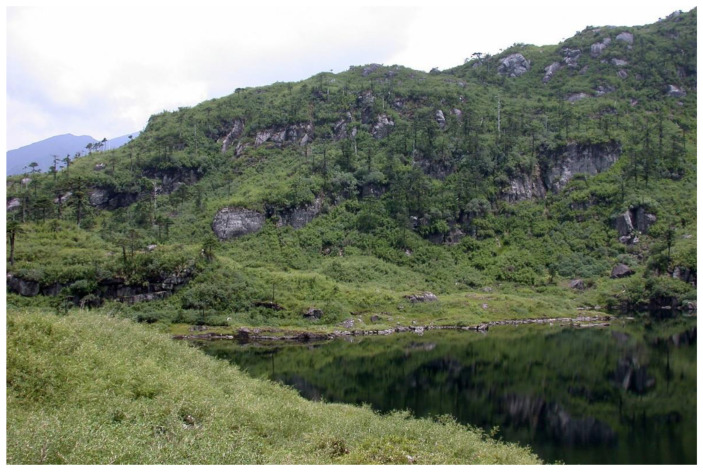
Collecting locality of *Marsupella praetermissa* Bakalin et *Vilnet* ***sp. nov.*** (Photo by D. Long, 2006).

Ecology: The species is known from the single specimen that was collected from granitic cliffs on the alpine lake shore (a small boulder under a shady dripping cliff) at an elevation of 3455 m a.s.l. (Figure 4, left corner). This is a primarily forestless landscape on gentle NE-facing slopes and a complex of lakes. The nearest forests are approximately 3 km downstream at the S-facing slopes at elevations of 3200–3300 m a.s.l.; however, scattered trees are present near the collecting locality. The associates within the specimens included *Riccardia* sp., *Gymnomitrion* sp. (belonging to the morphological group ‘*Apomarsupella’*), and *Sphenolobopsis pearsonii* (Spruce) R.M. Schust.

Comment: The distribution of the taxon could hardly be expected given the availability of only one record. Although the collecting locality is formally located in the Salween (Nu Jiang) River catchment, the collecting locality is only 500 m eastward from the pass to the Irrawaddy (Ayeyarwady) River catchment, the largest Myanmar River. Therefore, in a broad context, this is an area where several main river upper courses meet one another (Salween, Irrawaddy, Brahmaputra, and Mekong). Although strong differences in the species content between closely situated sites have been known here for over a century [11,12], the actual distribution of the taxon may be not very narrow, and additional records, including those from remote areas (around Sino-Himalaya), are highly possible.

Superficially, when assessing a small sample under the dissection microscope, the species is morphologically very similar to *M. stoloniformis*, which may explain the original misidentification of the specimen. The specimen was subjected to sequencing by Shaw et al. [4]. In the various topologies we attempted to combine, this specimen occupied an isolated position from the accession of *M. stoloniformis* from Vietnam that required further morphological investigation. Shortly thereafter, it was noted that even in dry conditions, the superficial morphology of the taxon was different from that of *M. stoloniformis* (both type in KYO and our vouchers from Vietnam). The leaves of *M. stoloniformis* are always (even when wet) appressed to the stem, such that the stem diameter is almost the same as the plant width. However, as described here, *M. praetermissa* has leaves that narrowly spread from the stem, even when the plants are dry. Another very distinctive feature that is evident under the dissecting microscope is the presence of small but regular underleaves—a feature that is unknown in *M. stoloniformis*. The third difference helps to distinguish the species from *M. stoloniformis* and is evident in the stem cross section in the microscope slide. *Marsupella praetermissa* has distinctly hyalodermatic outer cells with very thin walls, while the outer cells of *M. stoloniformis* are distinctly thick and hardly different from medullary cells.

**Figure 5:**
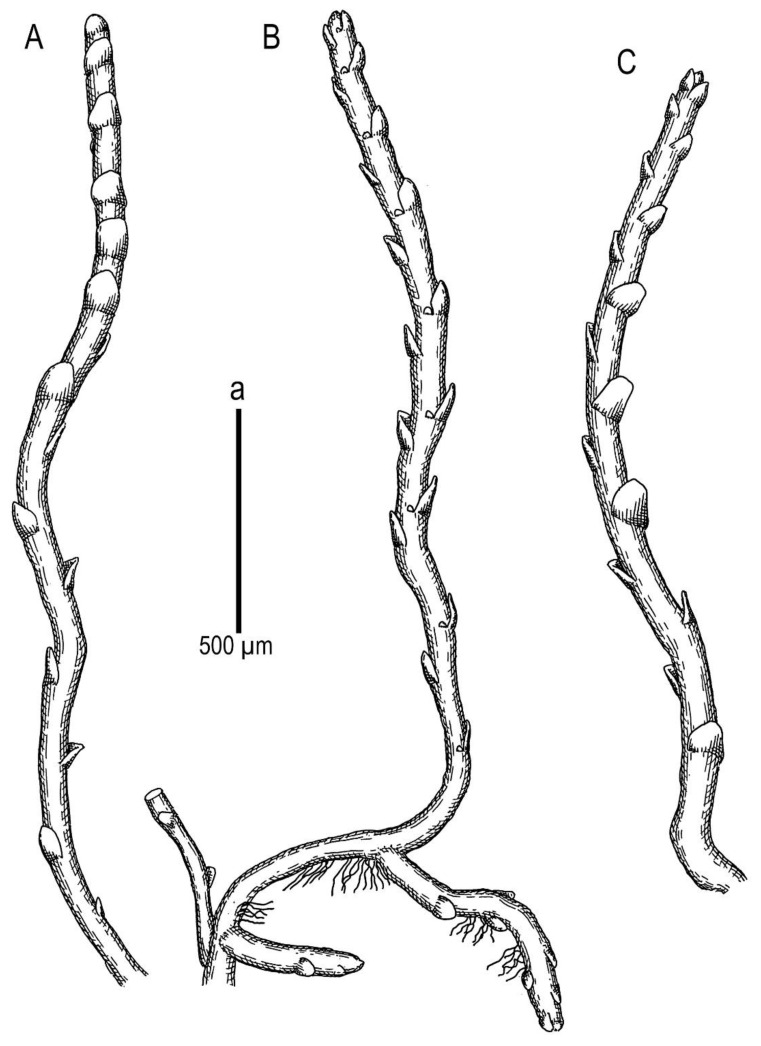
*Marsupella praetermissa* Bakalin et Vilnet ***sp. nov.***: (**A**) plant habit, fragment, lateral view; (**B**) plant habit, fragment, ventral view; (**C**) plant habit, fragment, dorsal view. Scales: a—500 µm for (**A**–**C**). All from Holotype Long 35742 (E).

**Figure 6:**
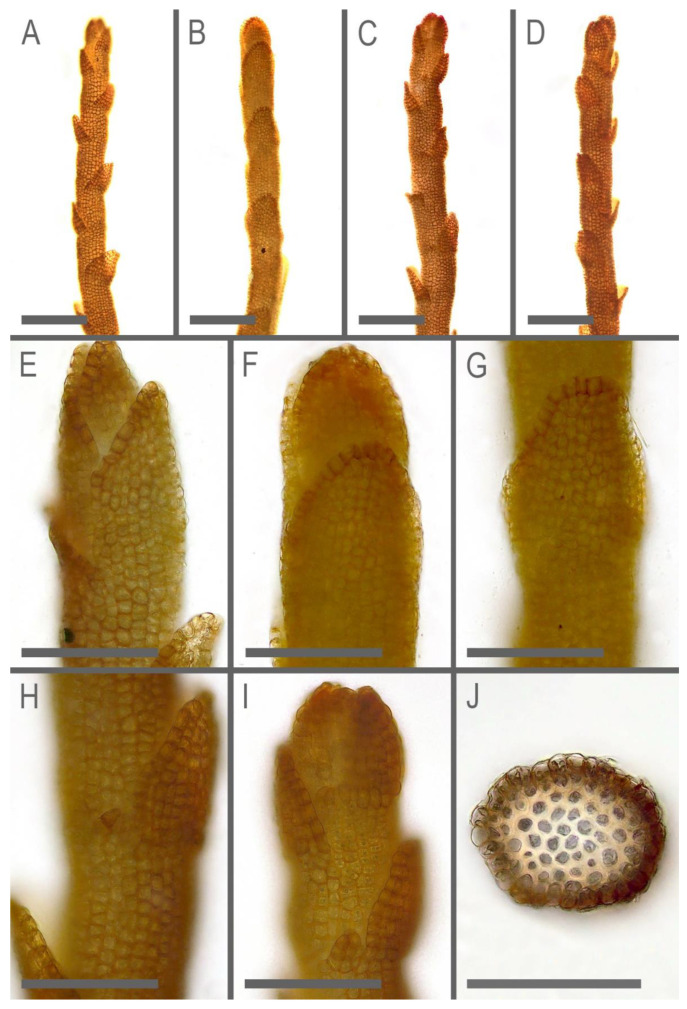
*Marsupella praetermissa* Bakalin et Vilnet ***sp. nov.***: (**A**) shoot, fragment, dorsal view; (**B**) shoot, fragment, lateral view; (**C**,**D**) shoot, fragment, ventral view; (**E**) shoot apex, dorsal view; (**F**) shoot apex, lateral view; (**G**) shoot fragment, lateral view; (**H**) shoot fragment, ventral view; (**I**) shoot apex, ventral view; (**J**) stem cross-section. Scales: 200 µm for (**A**–**D**); 100 µm for (**E**–**J**). All from Holotype Long 35742 (E).

Illustrations in present paper: Figure 5 and Figure 6.

*Marsupella taiwanica* Mamontov, Vilnet et Schäf.-Verw. ***sp. nov.***

Description. Plants in loose mats, small, *Cephaloziella*-like, more or less rigid, brown to blackish brown (when dry), sterile shoots 200–250 µm wide, up to 4 mm long; gynoecial sectors 500–650 µm wide. Rhizoids absent or very few, colorless to grayish. Stem rarely produces ventral branches originated from axils of underleaves, almost always with 1–2 subfloral ventral innovations; stem cross section usually slightly transversely elliptic, differentiated into outer and medullar cells. Outer layer cells 7–14 µm along margin, with brownish, thin, or slightly unequally thickened walls, with small to moderate concave trigones. Medullar cells rounded to elliptical, 7–15 µm in diameter, with concave to convex and often confluent trigones, walls thick, yellowish colored, sometimes with visible median lamella. Leaves remote, transversely inserted, obliquely spreading and subtransversely oriented, widely ovate, 120–200 µm wide, 110–165 µm long, 1.01–1.23 times wider than long, margins plane, with “keel” line nearly straight, divided to 0.28–0.35(0.39) of the leaf length, sinus acute-angled to rectangular, lobes subequal, triangular, 6–8 cells wide at base, acute to apiculate and sometimes hooked at apex, ending by 1–2 superimposed cells. Cells in the leaf middle and leaf lobes oblong to subisodiametric, 5–6-angled, 7–18 × 10–17(22) µm, walls thin to equally thickened, trigones small, indistinct, concave-triangular, cuticle smooth, marginal cells rectangular to 5-angled. Underleaves usually distinct, located near the base of leaf, small, subulate or spatuliform, consisting of 3–8 cells. Dioecious. Male plants not seen. Unfertilized perianths with subfloral innovations below, subgynoecial leaves in 2–3 pairs, larger than sterile leaves, 270–430 µm wide, 250–370 µm long, with subequal to strongly unequal lobes, outer margins in upper part and sinus margins sometimes narrowly reflexed. Unfertilized perianth onion-shaped, shorter than the bracts, ca 140–240 × 270–300 µm. Perigynium well developed, 0.4–0.66 of the length of the unfertilized perianth, bracts sheathing the perianth, with lobe apices decurved from perianth. Otherwise unknown.

Holotype: China, Taiwan Province, Nantou Co., Taroko National Park, roadside along Highway 8 between High Experimental Station/Visitor Center and Mt. Hehuan North & West Peak Trail (24°09.79′ N, 121°17.3′ E), 2970–3000 m a.s.l., on soil that is exposed to sunlight, 17 October 2016, A. Schäfer-Verwimp 37663 (MHA, duplicates in PRC (sub *Cephaloziella divaricata*), TAIE, JE, CAS, FR, TAIM, VBGI, KPABG). The specimen was mentioned as *Cephaloziella divaricata* as new to Taiwan by Schäfer-Verwimp et al. [13].

Other specimens that were examined (paratypes): China, Taiwan Province, Chiayi Co., Yushan National Park, along the 2.2 km trail from Paiyun Lodge to Yushan West Peak (23°27′59.1″ N 120°56′50.0″ E), 3415 m a.s.l., *Abies kawakamii* forest and *Yushania* bamboo understory, in a rock crevice that was exposed to sunlight, 25 October 2018, A. Schäfer-Verwimp 39136 (MHA, TAIE, JE, VBGI, KPABG, CAS, FR).

Ecology: Acidophilic meso-xerophyte. This is a rare and poorly known species, and the data on its ecology may be incomplete. *Marsupella taiwanica* occupies well-exposed habitats, including bare soil on roadsides and rock fissures in high mountains where it forms blackish-brown pure patches.

Comment: *Marsupella taiwanica* is most similar to several *Cephaloziella* species, especially *C. divaricata* (Sm.) Schiffn.; *C. grimsulana* (J.B. Jack ex Gottsche & Rabenh.) Lacout.; and *C. varians* (Gottsche) Steph., due to (1) small shoots with brownish coloration forming blackish brown patches; (2) remote and transversely inserted, erect spreading leaves; (3) the presence of regular underleaves. All the mentioned *Cephaloziella* species occasionally occur in sterile conditions. However, in patches of *Marsupella taiwanica*, fertile plants are also occasionally absent. These circumstances make it difficult to distinguish *M. taiwanica* from the aforementioned *Cephaloziella* species. Under sterile conditions, *M. taiwanica* can be distinguished from these species based on the following indirect features. In *Cephaloziella*, the sterile leaves are generally more deeply bilobed for 0.5–0.8 their length, especially near the shoot apex [14] (p. 36, Figure 498: 10). However, in *M. taiwanica*, the sterile leaves have a shallower sinus, usually ca. 0.3 of the leaf length. In *Cephaloziella*, the underleaves are mostly located on stems between the bases of opposite leaves cf. [14,15,16], whereas the underleaves are usually located very close to the base of one of the leaves in a leaf pair in *M. taiwanica*. The fertile (gynoecial) shoots of *M. taiwanica* easily differ from *Cephaloziella* based on the shape of the perianths (hidden within bracts, abruptly narrowed to the mouth), the presence of distinct perigynium, and the shape of the bracts (lobes acute to obtuse or rounded at the apex with shallow sinus and entire margins). The present species differs from all *Marsupella* based on its *Cephaloziella*-like habit. Another feature is the presence of regular underleaves shared within *Marsupella* with *M. praetermissa* that are strongly dissimilar in other morphological features.

**Figure 7:**
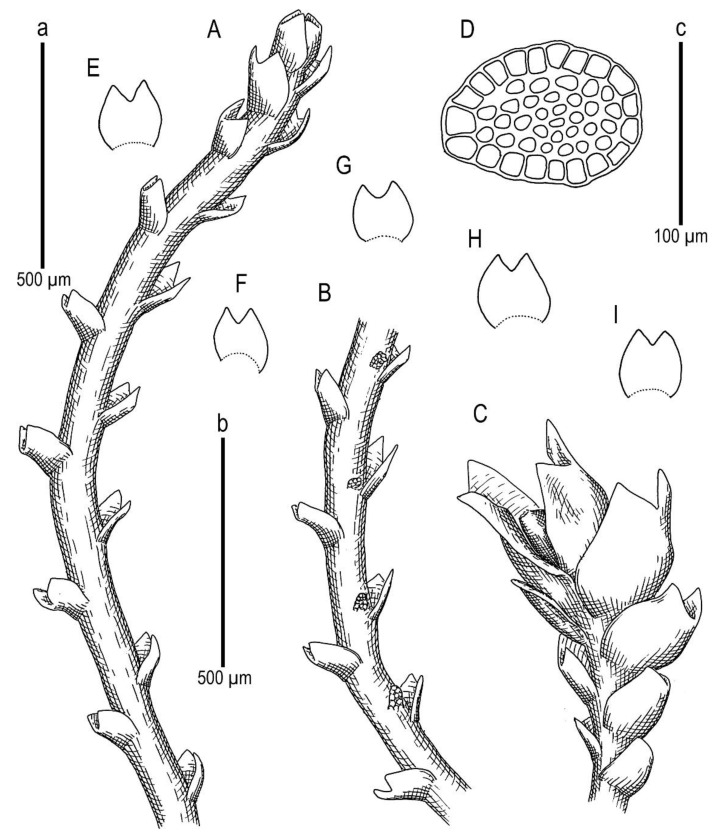
*Marsupella taiwanica* Mamontov, Vilnet et Schäf.-Verw. ***sp. nov.***: (**A**) shoot, fragment, dorsal view; (**B**) shoot, fragment, ventral view; (**C**) perianthous shoot, fragment; (**D**) stem cross-section; (**E**–**I**) leaves. Scale: a, b—500 µm (**A**–**C**,**E**–**I**); c—100 µm (**B**). All from Holotype Schäfer-Verwimp 37663 (MHA).

**Figure 8:**
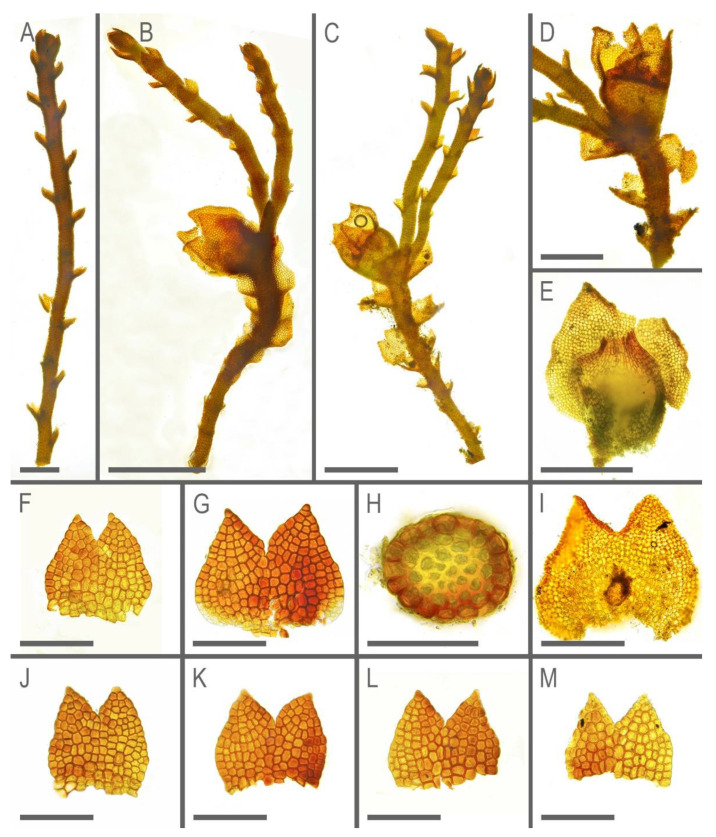
*Marsupella taiwanica* Mamontov, Vilnet et Schäf.-Verw. ***sp. nov.***: (**A**) sterile shoot, fragment, dorsal view; (**B)** perianthous plant, fragment, lateral view; (**C**) perianthous plant, fragment, ventral view; (**D)** perianthous shoot, fragment, ventral view; (**E**) perianth with female bract; (**F**,**G**,**J**–**M**) leaves; (**H**) stem cross-section; (**I**) female bract. Scales: 100 µm for (**F**–**H**,**J**–**M**); 200 µm for (**A**,**I**); 300 µm for (**D**,**E**); 500 µm for (**B**,**C**). All from Holotype Schäfer-Verwimp 37,663 (MHA).

Illustrations in present paper: Figure 7 and Figure 8.

### Identification Key to the Marsupella Sect. Stolonicaulon

**Figure 9:**
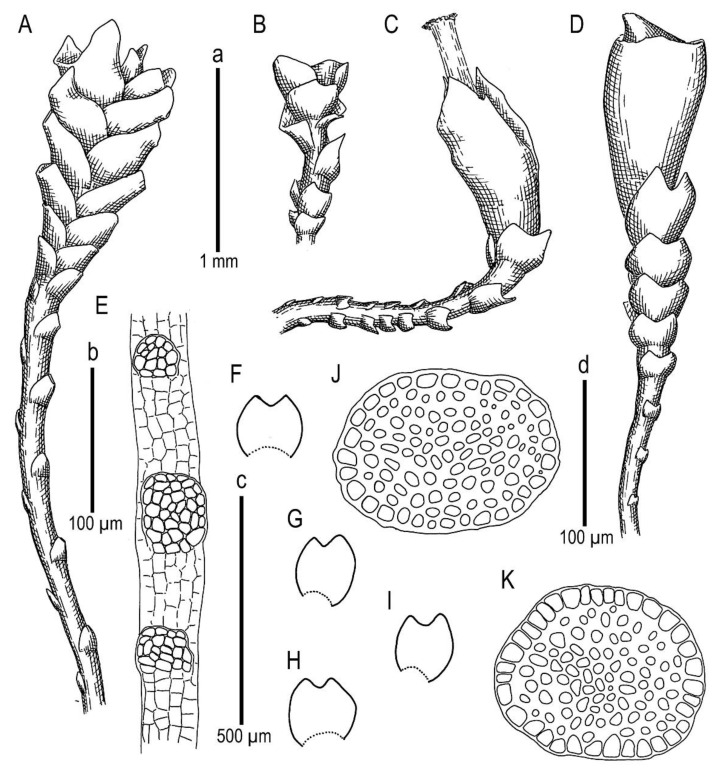
*Marsupella stoloniformis* N. Kitag.: (**A**) plant habit, fragment, dorsal view; (**B**) plant habit, fragment, dorsal view; (**C**) perianthous plant with seta; (**D**) perianthous plant, lateral view; (**E**) shoot with underleaves, fragment, ventral view; (**J**,**K**) stem cross-section; (**F**–**I**) leaves. Scale: a—1 mm for (**A**–**D**); b—100 µm for (**E**); c—0.5 mm for (**F**–**I**); d—100 µm for (**J**,**K**). (**A**,**D**,**F**–**J**) from V-11-11-17 (VBGI); (**B**,**C**,**E**,**K**) from Mizutani 2788 (KYO).

**Figure 10:**
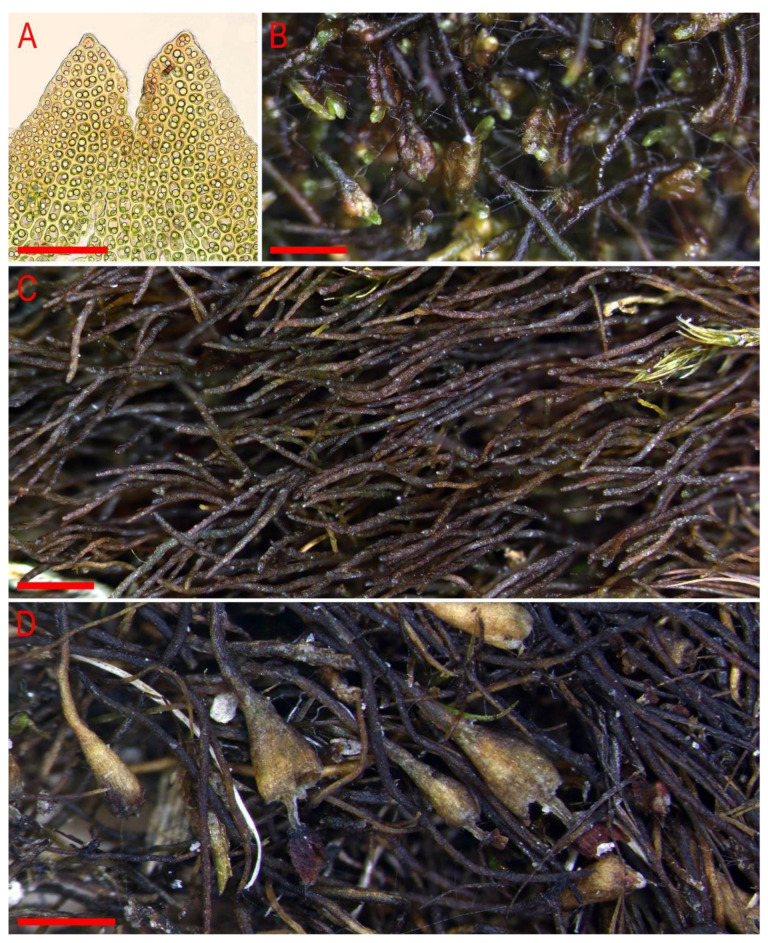
*Marsupella stoloniformis* N. Kitag.: (**A**) upper part of female bract; (**B**) mat, male plants, moist condition; (**C**) mat, sterile plants, moist condition; (**D**) mat, plants with perianths and open sporophytes, dry condition. Scale: 50 µm for (**A**); 1 mm for (**B**,**C**); 500 µm for (**D**). (**A**) from V-11-28-17 (VBGI); (**B**) from V-11-24-17 (VBGI); (**C**) from V-11-12-19 (VBGI); (**D**) from V-10-14-19 (VBGI).

**Figure 11:**
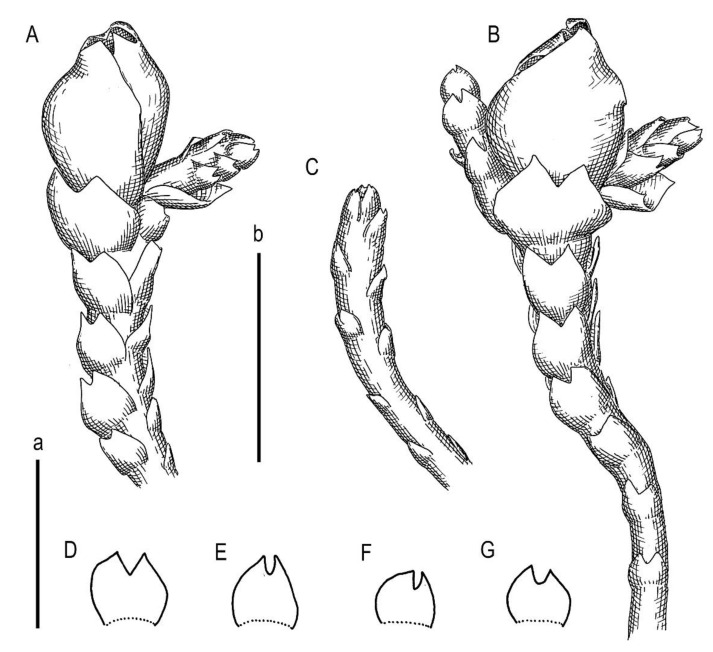
*Marsupella vermiformis* (R.M. Schust.) Bakalin et Fedosov: (**A**) perianthous shoot, fragment, lateral view; (**B**) perianthous shoot, fragment, lateral view; (**C**) sterile shoot, fragment, dorsal view; (**D**–**G**) leaves. Scale: a—500 µm for (**D**–**G**); b—500 µm for (**A**–**C**). All from Choi 120911 (VBGI).

1. Plants wiry, with strongly distanced appressed to the stem or narrowly spreading leaves, the stem diameter almost as large as the leaved shoot, leaves entire or shortly (less 1/5 in sterile shoots) divided … 2

1. Plants with contiguous to distanced, obliquely spreading, distinctly incised leaves 1/3–2/3 of the leaf length … 4

2. Plants with small, but regular underleaves, leaves narrowly spreading, stem cross section with hyalodermis … *M. praetermissa*

2. Plants free of underleaves, leaves appressed to the stem, stem cross section without hyalodermis … 3

3. Leaves entire to emarginate, if divided lobes rounded … *M. stoloniformis* (Figure 9 and Figure 10)

3. Leaves always divided by semi-crescentic sinus with lobes acute … *M. vermiformis* (Figure 11)

4. Plants similar to small *Anastrophyllum*, leaves contiguous, divided for 2/3 of the length, distinct hyalodermis present … *M. anastrophylloides*

4. Plants similar to *Cephaloziella*, leaves distant, divided to 1/3 of the leaf length, no distinct hyalodermis present (stem outer cells larger than inner, but merely thick-walled) … 5

5. Regular underleaves present [Taiwan] … *M. taiwanica*

5. Plants underleaf free [Venezuela, Brazil] … M. microphylla*

*—The taxon is known from a very few specimens; the concept of this taxon is derived from literature sources and needs critical testing.

## 5. Materials and Methods

### 5.1. Taxa Sampling

The study involved 6 specimens of *Marsupella* sect. *Stolonicaulon*: one voucher of *M. vermiformis* (with sequences that were obtained twice) from Jeju Island; one voucher that was identified as *M. stoloniformis* from North Vietnam; two accessions of unknown species from Taiwan (named below as *M. taiwanica*); one accession that was identified as *M. stoloniformis* from Yunnan Province of China (named below as *M. praetermissa*); and the specimen of unknown species from Hà Giang Province in North Vietnam (named below as *M. anastrophylloides*) (Table 2). All specimens were compared with each other, although they were so significantly different that we were occasionally inclined to compare them with the taxa from other sections, but not sect. *Stolonicaulon* itself.

The sequence data of ITS1-2 nrDNA and the *trn*L-F cpDNA data for the specimens of *M. vermiformis* (Republic of Korea) and *M. stoloniformis* (Vietnam) were published by Bakalin et al. [8], and the *trn*L-F and *trn*G-intron data for *M. stoloniformis* (China; as it is shown below, the identification was incorrect and belongs to *M. praetermissa* described below) were published by Shaw et al. [4]. In this study, ITS1-2 and *trn*L-F sequence data were obtained for two specimens from Taiwan and a single specimen from Vietnam, and the last *trn*G-intron was also sequenced. To reveal the phylogenetic affinity of these morphologically putative specimens, the alignment involving ITS1-2 and *trn*L-F nucleotide sequence data from twenty molecularly studied *Marsupella* species with representatives of four other Gymnomitriaceae genera was produced from GenBank accessions. *Eremonotus myriocarpus* from the phylogenetically related family Jungermanniaceae was placed as an outgroup.

### 5.2. DNA Isolation, PCR Amplification and DNA Sequencing

DNA was extracted using a DNeasy Plant Mini Kit (Qiagen, Germany) according to the manufacturer’s protocol. For the amplification and sequencing of ITS1-2 and *trn*L-F, the pairs of primers that were suggested by White et al. [17] and Taberlet et al. [18], respectively, were used. The *trn*G-intron of the Vietnamese specimens was obtained with primers from Shaw et al. [19]. A PCR was performed in a 20-µL volume using the following amplification cycles: 3 min at 94 °C; 30 cycles of 30 s 94 °C, 40 s 56 °C, 60 s 72 °C; and 2 min of final extension time at 72 °C. The amplified fragments were visualized on 1% agarose TAE gels by EthBr staining, purified using the Cleanup Mini Kit (Evrogen, Moscow, Russia), and then sequenced using the ABI Prism BigDye Terminator Cycle Sequencing Ready Reaction Kit (Applied Biosystems, Waltham, MA, USA) following the standard protocol that was provided for the 3100 Avant Genetic Analyser (Applied Biosystems, Waltham, MA, USA) (Genome Centre, Moscow, Russia).

### 5.3. Molecular Analysis

Two datasets for ITS1-2 and *trn*L-F loci were automatically aligned with the ClustalW option and then manually corrected in BioEdit 7.0.1 [20]. All positions were taken in estimation, and the absent data were coded as missing. Both datasets revealed congruence after preliminary phylogenetic analyses and were combined into a single ITS1-2+*trn*L-F alignment for subsequent estimations. The maximum likelihood (ML) with PhyML v.3.0 [21] and Bayesian approach with MrBayes v. 3.2.1 [22] were used to test phylogeny. The TN+I+G model was selected as the best-fit evolutionary model of nucleotide substitutions by ModelGenerator [23]. The 300 bootstrap replications were found to be sufficient for reaching BS convergence with a Pearson average of ρ100 = 0.995450 in RAxML v7.2.6 [24]. Thus, ML was run with the TN+I+G model with 300 bootstrap replicates, and the rate heterogeneity among the sites was modelled using a gamma distribution with four rate categories. For the Bayesian analysis, each partition of the combined alignment (ITS1-2, *trn*L-F) was separately assigned to the GTR+I+Г model, as suggested by the program creators, and gamma distributions were approximated using four rate categories. Two independent runs of the Metropolis-coupled ΜCMC were used to sample the parameter values in proportion to their posterior probability. Each run included three heated chains and one unheated chain, and two starting trees were chosen randomly. Chains were run for five million generations, and the trees were sampled every 100th generation. The software tool Tracer v.1.7.1—[25] revealed an effective sample size of 18,380.9242 and an autocorrelation time of 489.6489. The first 12,500 (25%) trees in each run were discarded as burn-in. Thereafter, 75,000 trees were sampled from both runs. The average standard deviation of split frequencies between the two runs was 0.001548. The Bayesian posterior probabilities were calculated from the trees that were sampled after burn-in. The majority rule (MJ) consensus tree was calculated after combining the runs minus burn-in of 25%, and the topology was illustrated with FigTree v. 1.4.4 (http://tree.bio.ed.ac.uk/software/figtree/, 4 June 2022) [26].

The sequence variability among the specimens of the section *Stolonicaulon* was evaluated as the *p*-distances for each DNA locus in Mega 11 [27] using the pairwise deletion option for counting gaps.

## 6. Conclusions

It was somewhat difficult even for a specialist to recognize the simple *Marsupella* in two of three newly described species and, even more so, to refer these taxa to the section *Stolonicaulon* without a molecular-genetic analysis. Indeed, after this work was performed, the genus *Marsupella* became more morphologically inconsistent, whereas the family Gymnomitriaceae itself became more balanced morphologically. Based on the newly described species of *Marsupella*, the subfamily Gymnomitrioideae evidenced distinct morphological connections in the presence of underleaves with an isolated subfamily Nardioideae. This work shows that even in “long-existing” genera, new species can be found that completely differ morphologically from previously known representatives. To speculate based on the obtained conclusions, the Sino-Himalayas and even the seemingly well-studied Taiwan Island may hide many undescribed species.

## Figures and Tables

**Table 1:** The values of *p*-distances, calculated for specimens from the section *Stolonicaulon* based on ITS1-2 and *trn*L-F nucleotide sequence data.

Species	*p*-Distances, ITS1-2/*trn*L-F, %					
	1	2	3	4	5	6
*M. vermiformis*, 1						
*M. vermiformis*, 2	0.4/0.0					
*M. stoloniformis*	4.0/4.2	3.6/4.2				
*M. taiwanica*, 1	3.8/5.5	3.4/5.5	2.5/3.9			
*M. taiwanica,* 2	3.7/5.7	3.4/5.7	2.5/4.2	0.1/0.3		
*M. praetermissa*	-/7.6	-/7.6	-/6.0	-/5.2	-/5.5	
*M. anastrophylloides*	4.4/8.9	4.1/8.9	3.5/7.3	3.4/7.0	3.5/7.3	-/3.9

**Table 2:** The list of tested specimens with voucher details and GenBank accession numbers. Newly generated accessions are marked in bold.

Taxon	Specimen Voucher	GenBank Accession Number	
		ITS1-2 nrDNA	*trn*L-F cpDNA
*Eremonotus myriocarpus* (Carrington) Lindb. & Kaal. ex Pearson	Russia: Karachaevo-Cherkessian Rep., N. Konstantinova, K446-6-05, 109,615 (KPABG)	EU791839	EU791716
*Gymnomitrion brevissimum* (Dumort.) Warnst.	Russia: Murmansk Prov., N. Konstantinova, G 8171 (KPABG)	EU791833	EU791711
*G. coralloides* Taylor ex Carrington	Norway: Svalbard, N. Konstantinova, K155-04, 110,103 (KPABG)	EU791826	EU791705
*Marsupella aleutica* Mamontov, Vilnet, Konstant. & Bakalin	USA: Alaska, Schofield, 103,958 (MO)	MH826408	MH822632
*M. anastrophylloides* Bakalin, Vilnet et Maltseva, sp. nov.	Vietnam: Hà Giang Prov., V. Bakalin & K. Klimova, V-15-6-20 (VBGI)	**OM480746**	**OM489480/** **OM489479** (*trn*G-intron)
*M. apertifolia* Steph.	Russia: Sakhalin Prov., V. Bakalin, K-79-2-15 (VBGI), 123,501 (KPABG)	MH539834	MH539891
*M. apiculata* Schiffn.	Norway: Svalbard, N. Konstantinova, K93-1-06, 111,840 (KPABG)	EU791819	EU791699
*M. aquatica* (Lindenb.) Schiffn.	Russia: Murmansk Prov., N. Konstantinova, 152-5-87, 6090 (KPABG)	EU791813	AF519201
*M. arctica* (Berggr.) Bryhn & Kaal.	Norway: Svalbard, N. Konstantinova, 128-04 (KPABG)	EU791815	EU791695
*M. boeckii* (Austin) Lindb. ex Kaal.	Russia: Murmansk Prov., N. Konstantinova, 367-2-00, 8184 (KPABG)	EU791816	EU791696
*M. bolanderi* (Austin) Underw.	USA: Santa Yen Mts. St. Barbara, 38,802 (KPABG), 1	MF521463	MF521475
*M. bolanderi*	USA: California Monterey CO, (KPABG), 2	MF521464	MF521476
*M. condensata* (Ångstr. ex C.Hartm.) Lindb. ex Kaal.	Russia: Kamchatka Terr., V. Bakalin, K-60-30-15 (VBGI)	MH539844	MH539901
*M. disticha* Steph.	Japan, Deguchi, Yamaguchi, Bryophytes of Asia 170 (2000) (KPABG)	EU791824	EU791703
*M. emarginata* (Ehrh.) Dumort.	Russia: Buryatia Rep., N. Konstantinova, 23-4-02, 104,411 (KPABG)	EU791811	EU791692
*M. funckii* (F. Weber & D. Mohr) Dumort.	Russia: Karachaevo-Cherkessian Rep., N. Konstantinova, K516-1-05, 109,804 (KPABG)	EU791820	EU791700
*M. koreana* Bakalin & Fedosov	Republic of Korea, KyongNam Province, V. Bakalin, Kor-23-18-15 (VBGI)	MH539850	MH539907
*M. patens* (N.Kitag.) Bakalin & Fedosov	Japan: Fukuoka Pref., V. Bakalin, J-7-26a-14 (VBGI)	MH539846	MH539903
*M. praetermissa* Bakalin, Vilnet et Long, sp. nov.	China: Yunnan Prov., D. Long, 35,742 (DUKE), published as Marsupella stoloniformis in Shaw et al. (2015)	No data	KF943111/KF942946 (*trn*G-intron)
*M. pseudofunckii* S.Hatt.	Japan: Yamanashi Pref., V. Bakalin, J-7-10-14 (VBGI)	MH539852	MH539909
*M. sphacelata* (Giesecke ex Lindenb.) Dumort.	Russia: Kemerovo Prov., N. Konstantinova, 65-1-00 (KPABG)	EU791821	AF519200
*M. sprucei* (Limpr.) Bernet	Russia: Kemerovo Prov., N. Konstantinova, 54-1-00, 101,850 (KPABG)	EU791823	HQ833031
*M. stoloniformis* N.Kitag.	Vietnam: Lao Cai Prov., V. Bakalin & K. Klimova, V-11-11-17 (VBGI)	MH539859	MH539916
*M. subemarginata* Bakalin & Fedosov	Japan: Yamanashi Pref., V. Bakalin, J-89-31-15 (VBGI), 123,468 (KPABG)	MH539836	MH539893
*M. taiwanica* Mamontov, Vilnet et Schäf.-Verw., sp. nov.	China: Taiwan, Nantou Co., A. Schäfer-Verwimp 37,663 (MHA, TAIE, JE, VBGI), 123,642 (KPABG), 1	**OM509627**	**OM515126**
*M. taiwanica* Mamontov, Vilnet et Schäf.-Verw., sp. nov.	China: Taiwan, Chiayi Co., A. Schäfer-Verwimp 39,136 (MHA, TAIE, JE, VBGI), 123,545 (KPABG), 2	**OM509628**	**OM515127**
*M. tubulosa* Steph.	Russia: Kamchatka Terr., V. Bakalin, K-66-7-15 (VBGI)	MH539860	MH539917
*M. vermiformis* (R.M.Schust.) Bakalin & Fedosov	Republic of Korea: Jeju Prov., S. Choi, 120,911 (VBGI), 1	MH539857	MH539914
*M. vermiformis*	Republic of Korea, Jeju Prov., S. Choi, 120911-1 (VBGI) 2	MH539858	MH539915
*M. vietnamica* Bakalin & Fedosov	Vietnam: Lao Cai Prov., V. Bakalin, V-2-101-16 (VBGI)	MH539862	MH539919
*M. yakushimensis* (Horik.) S.Hatt.	Republic of Korea: Gangwon Prov., S. Choi, 8347 (VBGI)	MH539864	MH539921
*Nardia compressa* (Hook.) Gray	Canada: British Columbia, N. Konstantinova, A 97/1-95 (KPABG)	EU791837	AF519188
*Prasanthus suecicus* (Gottsche) Lindb.	Norway: Svalbard, N. Konstantinova, K121-5-06, 111,821 (KPABG)	EU791825	EU791704

## Data Availability

Not applicable.

## References

[1] Kitagawa N. (1967). *Marsupellae* of Mt. Kinabalu, North Borneo. J. Hattori Bot. Lab..

[2] Schuster R.M. (1996). Studies on antipodal hepaticae. XII. Gymnomitriaceae. J. Hattori Bot. Lab..

[3] Vilnet A.A., Konstantinova N.A., Troitsky A.V. (2010). Molecular insight on phylogeny and systematics of the Lophoziaceae, Scapaniaceae, Gymnomitriaceae and Jungermanniaceae. Arctoa.

[4] Shaw B., Crandall-Stotler B., Váňa J., Stotler R.E., von Konrat M., Engel J.J., Davis E.C., Long D.G., Sova P., Shaw A.J. (2015). Phylogenetic relationships and morphological evolution in a major clade of leafy liverworts (phylum Marchantiophyta, order Jungermanniales): Suborder Jungermanniineae. Syst. Bot..

[5] Vana J., Söderström L., Hagborg A., Von Konrat M. (2014). Notes on Early Land Plants Today. 60. Circumscription of Gymnomitriaceae (Marchantiophyta). Phytotaxa.

[6] Váňa J., Söderström L., Hagborg A., von Konrat M.J., Engel J.J. (2010). Early Land Plants Today: Taxonomy, systematics and nomenclature of Gymnomitriaceae. Phytotaxa.

[7] Bakalin V., Choi S.S., Park S.J. (2021). Revision of Gymnomitriaceae (Marchantiophyta) in the Korean Peninsula. PhytoKeys.

[8] Bakalin V.A., Fedosov V.E., Fedorova A.V., Nguyen V.S. (2019). Integrative taxonomic revision of Marsupella (Gymnomitriaceae, Hepaticae) reveals neglected diversity in Pacific Asia. Cryptogam. Bryol..

[9] Mamontov Y.S., Vilnet A.A., Konstantinova N.A., Bakalin V.A. (2019). Two new species of Gymnomitriaceae (Marchantiophyta) in the North Pacific. Bot. Pac..

[10] Schuster R.M. (1974). The Hepaticae and Anthocerotae of North America East of the Hundredth Meridian. III.

[11] Ward F.K. (1921). The Mekong-Salween Divide as a geographical barrier. Geogr. J..

[12] Ward F.K. (1925). Sino-Himalaya. Nature.

[13] Schäfer-Verwimp A., Winter G., Hsu Y.C., Lin S.-H., Yang J.-D., Yao K.-Y. (2019). New and interesting bryophyte records for Taiwan. BryoString.

[14] Schuster R.M. (1980). The Hepaticae and Anthocerotae of North America East of the Hundredth Meridian. IV.

[15] Paton J.A. (1999). The liverwort flora of the British Isles.

[16] Damsholt K. (2002). Illustrated Flora of Nordic Liverworts and Hornworts.

[17] White T.J., Bruns T.D., Lee S.B., Taylor J.W., Innis M.A., Gelfand D.H., Snisky J.J., White T.J. (1990). Amplification and direct sequencing of fungal ribosomal RNA genes for phylogenetics. PCR Protocols: A Guide to Methods and Applications.

[18] Taberlet P., Gielly L., Guy Pautou G., Bouvet J. (1991). Universal primers for amplification of three non-coding regions of chloroplast DNA. Pl. Molec. Biol..

[19] Shaw J., Lickey E.B., Beck J., Farmer S.B., Liu W., Miller J., Siripun K.C., Winder C., Schilling E.E., Small R.L. (2005). The tortoise and the hare II: Relative utility of 21 noncoding chloroplast DNA sequences for phylogenetic analysis. Am. J. Bot..

[20] Hall T.A. (1999). BioEdit: A user-friendly biological sequence alignment editor and analysis program for Windows 95/98/NT. Nucleic Acids Symp. Ser..

[21] Guindon S., Dufayard J.F., Lefort V., Anisimova M., Hordijk W., Gascuel O. (2010). New Algorithms and Methods to Estimate Maximum-Likelihood Phylogenies: Assessing the Performance of PhyML 3.0. Syst. Biol..

[22] Ronquist F., Teslenko M., van Der Mark P., Ayres D.L., Darling S., Höhna S., Larget B., Liu L., Suchard M.A., Huelsenbeck J.P. (2012). MrBayes 3.2: Efficient Bayesian phylogenetic inference and model choice across a large model space. Syst. Biol..

[23] Keane T.M., Creevey C.J., Pentony M.M., Naughton T.J., Mcinerney J.O. (2006). Assessment of methods for amino acid matrix selection and their use on empirical data shows that ad hoc assumption for choice of matrix are not justified. BMC Evol. Biol..

[24] Stamatakis A. (2006). RAxML-VI-HPC: Maximum Likelihood-based Phylogenetic Analyses with Thousands of Taxa and Mixed Models. Bioinformatics.

[25] Rambaut A., Drummond A.J., Xie D., Baele G., Suchard M.A. (2018). Posterior summarisation in Bayesian phylogenetics using Tracer 1.7. Syst. Biol..

[26] FigTree v. 1.4.4. http://tree.bio.ed.ac.uk/software/figtree/.

[27] Tamura K., Stecher G., Kumar S. (2021). MEGA11: Molecular Evolutionary Genetics Analysis version 11. Molec. Biol. Evol..

